# The Impact of Pulmonary Rehabilitation in Acute Exacerbation of Chronic Obstructive Pulmonary Disease (COPD): A Case Report

**DOI:** 10.7759/cureus.55537

**Published:** 2024-03-04

**Authors:** Vaishnavi R Waghe, Lajwanti Lalwani, Priyanka K Chilhate

**Affiliations:** 1 Department of Cardiovascular and Respiratory Physiotherapy, Ravi Nair Physiotherapy College, Datta Meghe Institute of Higher Education and Research, Wardha, IND

**Keywords:** physical therapy, dyspnea, pulmonary rehabilitation, chronic obstructive pulmonary disease, acute exacerbation

## Abstract

This study focuses on a 56-year-old male laborer who presented to the respiratory department with grade III dyspnea persisting for 20 days, aggravated in the mornings, accompanied by chest pain over the last two days. The patient reported a productive cough producing yellowish sputum for 15 days and an ongoing fever during this period. With a two-year medical history of seasonal bronchial asthma, the patient had been using an inhaler three times daily for the past month. Additionally, a 20-year history of smoking, averaging five cigarettes per day, was disclosed. Investigations revealed hyperinflation of the lungs on X-ray, indicative of an acute exacerbation of chronic obstructive pulmonary disease (AECOPD). The patient was prescribed a four-week pulmonary rehabilitation protocol, incorporating physiotherapy. Baseline assessments were conducted using outcome measures such as pulmonary function test (PFT), functional independence measure (FIM), and six-minute walk distance (6MWD) before initiating treatment to evaluate the patient's performance. Following the prescribed pulmonary rehabilitation regimen, notable improvements were observed in PFT, FIM, and 6MWD. These findings underscore significant enhancements in exercise tolerance and overall functional capacity. The results suggest that a structured pulmonary rehabilitation program can lead to meaningful clinical benefits in individuals experiencing AECOPD, particularly when tailored to individual patient needs and characteristics.

## Introduction

Acute exacerbations of chronic obstructive pulmonary disease (AECOPD) manifest as a worsening of respiratory symptoms, cough, and/or sputum beyond the patient's typical daily experience, necessitating adjustments in medication [1]. Chronic obstructive pulmonary disease (COPD) is characterized by progressive lung impairment [2]. Infectious etiologies associated with AECOPD stem from a diverse array of bacteria and viruses. External factors such as smoking, air pollution, and weather changes influence the frequency of AECOPD [3]. Annually, 25% of COPD patients require acute hospitalization due to exacerbations [4]. While both infectious and noninfectious factors contribute to AECOPD, it is noteworthy that up to 30% of cases have an unknown cause [5]. Periodic acute exacerbations of respiratory dysfunction punctuate the course of COPD, a chronic ailment, often serving as primary drivers for hospitalization or mortality [6]. In the vast majority of patients, COPD manifests as a progressive condition characterized by declining lung function over time. This decline is compounded by the natural deterioration of pulmonary function associated with advancing age. Undiagnosed COPD in its early stages, especially among symptomatic individuals, tends to advance to more severe disease stages. This progression significantly impacts health-related quality of life (HRQOL), escalates healthcare costs, and necessitates greater utilization of healthcare resources [7].

The 2019 Global Initiative for Chronic Obstructive Lung Disease (GOLD) report underscores the importance of exercise-based respiratory rehabilitation as a cornerstone in patient care [8,9]. Based on current research, a combination of anaerobic and aerobic exercises is recommended [10]. Reports indicate that 77% of United Kingdom physiotherapists routinely administer cardiorespiratory physiotherapy interventions to hospitalized COPD patients experiencing acute exacerbations [11]. According to the official American Thoracic Society/European Respiratory Society statement, pulmonary rehabilitation is a “comprehensive intervention based on a thorough patient assessment followed by patient-tailored therapies that include, but are not limited to, exercise training, education, and behavior change, designed to improve the physical and psychological condition of people with chronic respiratory disease and to promote long-term adherence to health-enhancing behaviors” [8]. According to reports, physical therapy (PT) helps COPD patients by minimizing dyspnea and enhancing lung function, respiratory muscle strength, and overall quality of life [12,13]. A Hong Kong randomized controlled trial (RCT) revealed that while an early eight-week rehabilitation course after AECOPD improved quality of life (QOL) for up to six months, it had no effect on healthcare utilization beyond a year [14].

## Case presentation

A 56-year-old male laborer arrived at the Acharya Vinoba Bhave Rural Hospital respiratory department with grade III dyspnea, which had been present for 20 days and was worse in the mornings. The patient also complained of experiencing chest pain during the previous two days, along with a productive cough that produced yellowish sputum and persisted for 15 days, accompanied by a persistent fever throughout the same period of time. The patient had a two-year medical history of seasonal variability related to bronchial asthma, and for the past month, he had been using an inhaler three times a day. The patient also revealed that he had smoked for 20 years, averaging five cigarettes a day. Upon investigation, the results of the blood and urine tests were found to be normal, and an X-ray finding showed hyperinflation of the lungs suggestive of AECOPD. The patient was advised for hospital admission, during which he received intravenous administration of hydrocortisone 100 mg along with intravenous injection of furosemide 20 mg. Additionally, he was prescribed oral medications including clopidogrel 300 mg, aspirin 300 mg, and atorvastatin 80 mg, and asked to seek additional care in physiotherapy. The patient was conscious and oriented with a pulse rate of 130 beats per minute, 26 respirations per minute, blood pressure of 116/72 mmHg, weight of 54 kg, and oxygen saturation of 93% after receiving three liters of oxygen via nasal cannula.

Clinical findings

**Figure 1:**
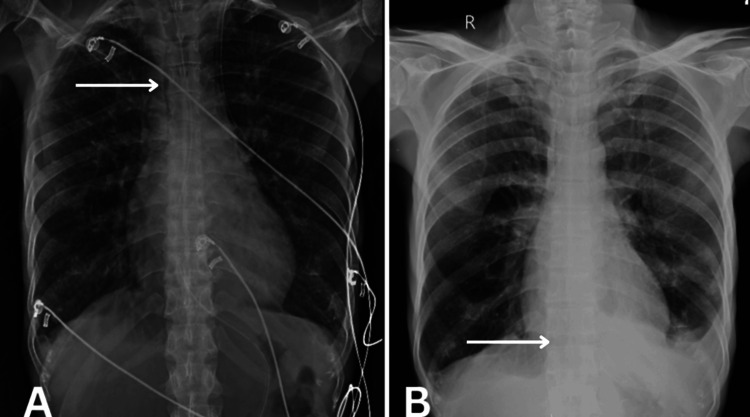
X-ray showing (A) increased mediastinal widening and (B) mild obliteration of angle

Informed consent was obtained before the patient was examined. Upon examination, the patient was observed to be cooperative and aware of time, place, and people. Breathlessness with the use of accessory muscles, grade III, was present according to the Modified Medical Research Council (MMRC) scale. Upon palpation at the axillary, nipple, and xiphisternum levels, the results for chest expansion were 1 cm, 2 cm, and 2 cm, respectively. There was a hyper-resonant note on percussion at the middle and lower zones. Bilateral wheezing was positive (left > right) with equal bilateral air entry on auscultation. The chest X-ray of the patient is shown in Figure 1.

Therapeutic intervention

The patient's rapid recovery was facilitated by the interdisciplinary approach to his care with pulmonary rehabilitation. Our primary objective was to alleviate dyspnea and improve hypoxemia by augmenting gaseous exchange and lung air entry. The pulmonary rehabilitation administered to the patient is depicted in Table 1.

**Table 1:** Respiratory intervention protocol AECOPD: Acute exacerbation of chronic obstructive pulmonary disease

Problem list	Goals	Treatment (four weeks)
Patient education	To educate about the AECOPD condition and the importance of physiotherapy treatment for the same	The patient was told about his condition and how physiotherapy helps recover faster and was explained the treatment protocol
Exertional dyspnea	To reduce dyspnea while working	Pursed lip breathing (10 repetitions, two sets), dyspnea relieving position
Use of accessory muscles	Provide relaxation to muscles	Relaxation techniques, diaphragmatic breathing (10 repetitions, two sets)
Accumulation of secretions	Removal of secretions	Nebulization for 10 minutes with duolin medication, active cycle of breathing technique (three cycles)
Reduced endurance	To improve exercise tolerance and general mobility, and improve quality of life	Spot marching (10 repetitions, two sets), ambulation (five rounds, i.e., 300 m), static cycling initiated with no resistance (2 min)

**Figure 2:**
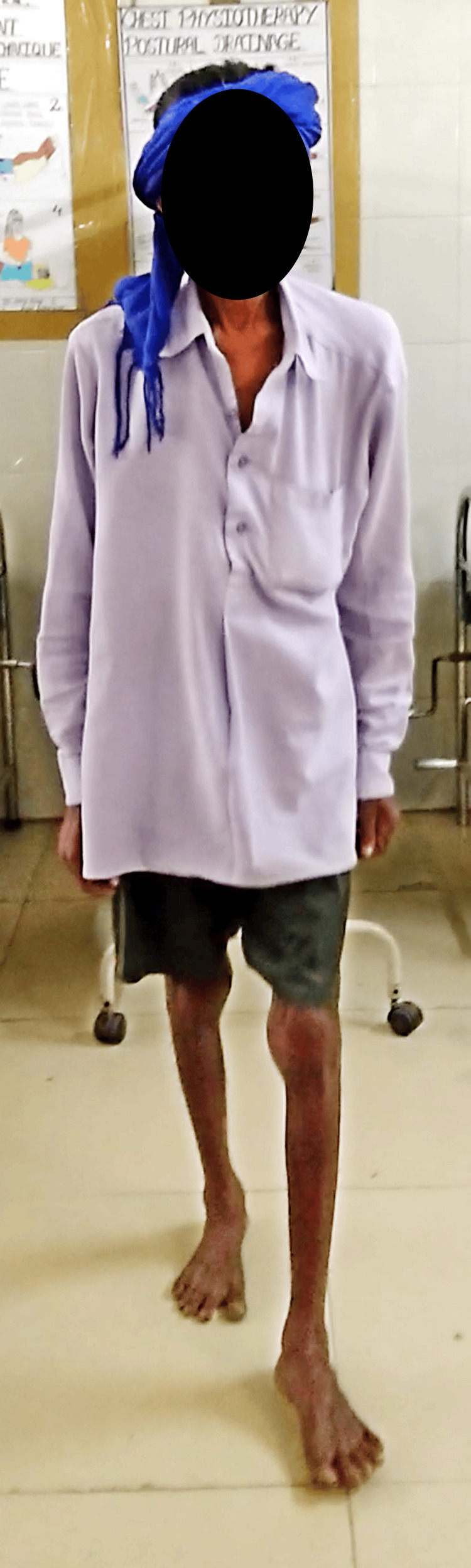
Patient ambulating in the ward

**Figure 3:**
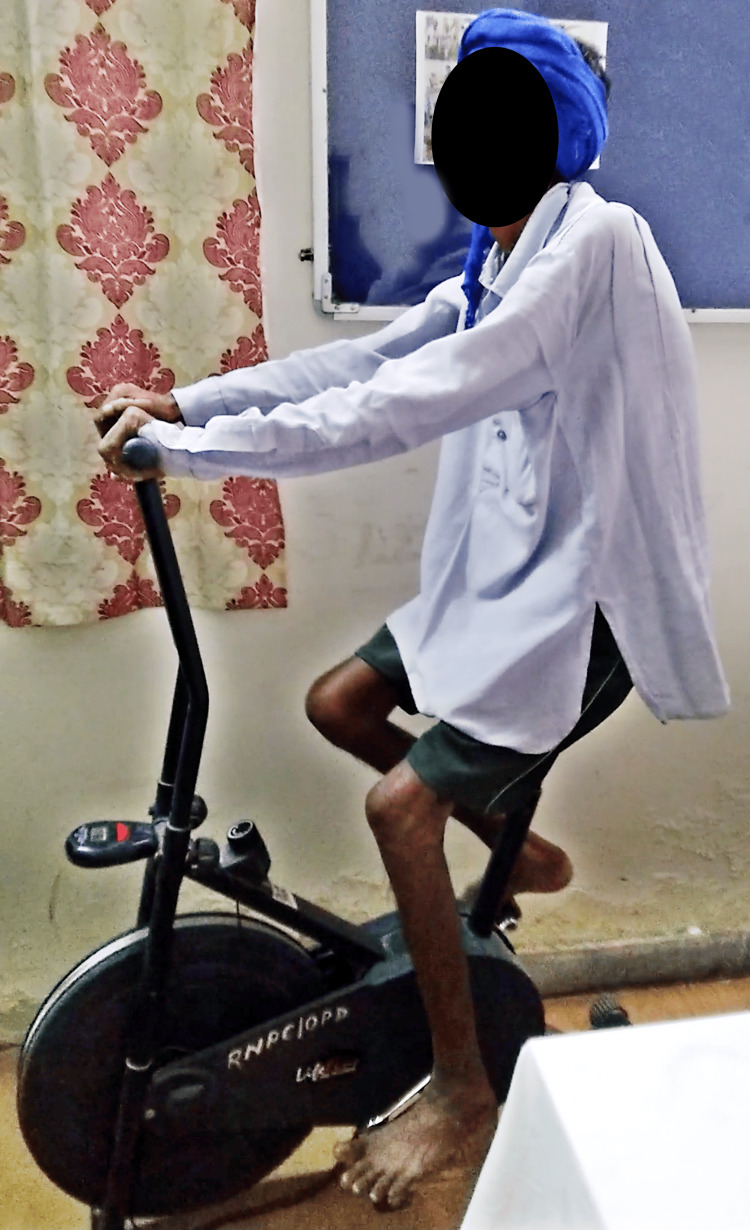
Patient performing static cycling

Progression was done to improve the strength, balance, and flexibility with functional activities by increasing the sets, repetitions, and resistance, or by decreasing the rest time. Patient performing exercises, i.e., ambulation and static cycling, were given for flexibility and to improve exercise capacity as a part of aerobic exercise, as shown in Figure 2 and Figure 3, respectively.

Follow-up and outcomes

Table 2 shows the outcome measures on assessment, discharge, and after two weeks during follow-up. The pulmonary function test (PFT) reveals a mild obstruction upon follow-up, likely attributed to enhanced airway clearance and improved exercise tolerance. Furthermore, the six-minute walk test (6MWT) demonstrates notable enhancement, with a substantial increase of 270 meters in distance covered within a span of four weeks.

**Table 2:** Follow-up and outcome measures FVC: Forced vital capacity; FEV1: Forced expiratory volume in the first second; O_2_: Oxygen

Outcome	On assessment	On discharge	On follow-up
Pulmonary function test	FVC: 01.02L	FVC: 01.24L	FVC: 01.54L
	FEV1: 00.41L	FEV1: 00.58L	FEV1: 00.71L
	FEV1/FVC: 40.20%	FEV1/FVC: 57.26%	FEV1/FVC: 72.28%
Functional independence measure	82	98	112
Six-minute walk distance	70 m with 3L of O_2_	210 m without O_2_	340 m without O_2_

## Discussion

This case emphasizes the effects of pulmonary rehabilitation in patients experiencing an AECOPD. Rehabilitation enhances exercise tolerance, respiratory function, and both the physical and educational aspects. The research conducted by Carlos A. Camillo underscores the pivotal role of the six-minute walk distance (6MWD) as a prognostic indicator in evaluating the mortality risk among individuals diagnosed with COPD. Additionally, it contributes novel insights by revealing that patients who do not exhibit a minimum improvement of 30 meters in 6MWD, coupled with those experiencing suboptimal rehabilitation outcomes, demonstrate diminished probabilities of survival over a five-year follow-up period [15]. In individuals afflicted with advanced COPD, the initial 6MWD emerged as a prognostic indicator for survival. The cumulative survival rate after a three-year period stood at a mere 58%, with a notably dismal outcome observed in those with an initial walk distance of less than 150 meters, where the survival rate plummeted to 34% [16].

A recent proposition has emerged suggesting that the functional independence measure (FIM) could potentially serve as a valuable outcome measure for individuals engaging in pulmonary rehabilitation programs following an AECOPD [17]. Moreover, its applicability extends to individuals undergoing endotracheal intubation and subjected to mechanical ventilation within the confines of intensive care units (ICUs) [18]. Subsequent to pulmonary rehabilitation in patients diagnosed with COPD, Giulia Montagnani elucidates a statistically noteworthy enhancement in the mean FIM global score, progressing from 97.4 with a standard deviation (SD) of 27.5 to 102.5 (SD=25.7) [19].

Spirometry stands as the definitive diagnostic gold standard for COPD. In symptomatic individuals, the application of spirometry serves as a pivotal tool in discerning the etiology of respiratory symptoms, distinguishing between those attributable to respiratory pathology and those arising from alternative conditions [20]. Individuals presenting with a more compromised disease status, characterized by a combination of lower forced expiratory volume in one second (FEV1), increased hyperinflation, and diminished exercise capacity, demonstrate a more substantial improvement in endurance exercise capacity [21]. Within our study, outcome measures serve as essential tools for assessing patient performance, thereby facilitating a comprehensive evaluation that correlates with significant improvements in exercise tolerance and functional capacity of patients. The findings indicate that the patient has demonstrated notable advancements in pulmonary function, as evidenced by improvements in the PFT. Additionally, there has been an observed increase in ambulatory distance during the 6MWT, suggesting enhanced functional capacity and endurance.

## Conclusions

This study concludes that the enhancements observed in airway clearance, exercise capacity, and overall functional outcomes underscore the effectiveness of a comprehensive rehabilitative approach in alleviating the severity and consequences associated with acute COPD exacerbations. Notably, our study reveals a prompt and significant improvement within the four-week rehabilitation period, particularly in enhancing functional capacity, managing symptoms, and improving overall quality of life. These findings emphasize the potential of early and intensive pulmonary rehabilitation interventions to significantly impact the trajectory of COPD exacerbations and improve patient outcomes.
